# Electronic News Dataset for Native Advertisement Detection

**DOI:** 10.1038/s41597-024-04341-6

**Published:** 2025-06-20

**Authors:** Brian Rizqi Paradisiaca Darnoto, Daniel Siahaan, Diana Purwitasari

**Affiliations:** 1 Informatics Department, Institut Teknologi Sepuluh Nopember, Surabaya, Indonesia; 2 Informatics, Universitas Jember, Jember, Indonesia

**Keywords:** Computer science, Information technology

## Abstract

Native advertising has rapidly evolved into a predominant online marketing strategy, adequately blending with editorial content to the extent that readers often remain unaware they are involved with sponsored material. This integration, spanning diverse formats like text articles, videos, and social media posts, enriches its application but concurrently escalates the detection challenge. The nuanced identification and comprehension of native advertising necessitates advanced methodologies, underpinned by datasets that are both relevant and expansive, ensuring transparency in online environments. This paper elucidates a systematic approach to data collection and annotation, aiming to construct a specialized dataset poised to significantly enhance native ads detection efforts. Through the meticulous aggregation of news from six mainstream electronic news portals in Indonesia, the study extends beyond mere identification of native ads to also encompass four additional implicit characteristics typically associated with such content. The resulting, carefully annotated dataset emerges as an invaluable resource, promising to propel the development and evaluation of sophisticated native ads detection algorithms. This advancement holds the potential to augment transparency within online advertising and contribute to the clearer delineation between pure editorial content and sponsored material.

## Background & Summary

Native advertising (native ads) represents a form of integrated commercial content that has been notably effective in engaging online consumers. A significant factor in its effectiveness is that consumers frequently do not perceive its promotional intent^1^. These ads often take the form of articles, videos, or reviews, and are difficult to distinguish from the original content. Native ads first appeared in 2012 and have since become a popular form of digital advertising. They offer several advantages compared to other forms of digital advertising. Firstly, native ads are more likely to be seen and clicked on than banner or pop-up ads. Secondly, native ads are more likely to be remembered by users. Thirdly, native ads are more likely to generate conversions.

Youn^2^ reveals three main themes regarding reader interaction with native ads: persuasive knowledge, positive and negative experiences, and avoidance responses. While native ads offer numerous advantages, they often provoke strong negative reactions, leading to backlash and ad avoidance. Consequently, many people are unaware that they are interacting with content that is sponsored and paid for^3^. Wojdynski’s^4^ significant effects of native ads indicate that this form of advertising can deceive customers unaware of its nature. In this context, the deception helps mitigate the negative assessments that arise when consumers realize the content is actually an advertisement. Taylor^5^ suggests that the primary problem with native ads is the lack of explicit disclosure signalling that the content is promotional.

Cornwell^6^ demonstrates that native ads often have few signs warning readers that they are advertisements, leading readers to struggle in recognizing them. Native advertisements possess both explicit and implicit characteristics. The explicit features are typically identifiable through labels such as “sponsored content” or “advertorial” within the news details. Additionally, they are often found in URLs categorized under “advertorial” or similar terms. Amazeen^7^ explains that the use of clear labelling becomes the only feature consistently distinguishing sponsored content from editorial content. The explicit characteristics of native ads are easily recognized, unlike their implicit traits. Native ads are designed to influence user behaviour. They frequently employ persuasive techniques, such as calls to action or customer testimonials. Native advertisements generally maintain a positive or neutral tone, avoiding the use of aggressive or manipulative language. Native ads should reflect the values and image of the advertised company or product. They must align with the messaging and communication strategies of the company or product. Native ads present a singular perspective, conveying messages that are specific and relevant to the targeted audience.

In the field of text processing, the availability of datasets is a key factor affecting the performance of developed systems. Wang^8^ introduced LIAR, an innovative public dataset for fake news detection. This dataset contains 12,000 manually labeled short statements collected over twenty-five years from POLITIFACT.COM, including analytical reports and links to source documents for each entry. Ahmed^9^ gathered datasets from real-world sources, comprising both genuine and fake news. Authentic articles were sourced from Reuters.com, while the fabricated entries were drawn from a fake news dataset on kaggle.com, totalling 12,600 articles from each category, all exceeding 200 characters in length. Fawaid^10^ undertook a study aimed at curbing the proliferation of fake news in the Indonesian language, using a dataset acquired from the news portal turnbackhoax.id, which specializes in identifying fake news.

Datasets of native advertisements utilizing the Indonesian language remain unavailable. However, a dataset of native ads in Slovak is held by Andicsova’s research^11^. Andicsova engaged in the detection of native ads by leveraging explicit characteristics on Slovak electronic news portals. This was achieved through web scraping from three Slovak news sites. The subcategories utilized were authentic articles most frequently mimicked by native ads, such as celebrity news or health news. The total dataset comprised 3,000 original articles and 2,455 native advertising articles. The publication dates of the articles from the websites ranged from November 2006 to March 2021. Andicsova did not employ annotators, as the news clearly contained native advertisements.

Based on prior research, we have developed the Native Ads Dataset to identify native advertisements within Indonesian online news outlets. 12,088 news stories from six online news sources are included in the collection. The six news portals were selected based on particular standards relevant to our study’s objective of identifying native ads. These factors include the portals’ level of popularity, the variety of the content they post, and the possibility that these platforms may either overtly or covertly use native advertising. We also took into account how easily accessible the data from these portals was utilizing legally compliant scraping methods. A well-rounded sample of the different kinds of news information accessible on online platforms is represented by the six chosen portals, which is essential for guaranteeing data variety. Every article has undergone a thorough validation procedure and has been painstakingly annotated, guaranteeing high-quality data integrity. The portals chosen for this study offer thorough coverage of a wide range of subjects, demographics, and geographical areas, representing a varied cross-section of Indonesia’s most significant digital media platforms. In addition to labels identifying native advertisements, the dataset features four labels that capture the implicit features of native ads. These four characteristics are: sentiment labels, persuasive labels, labels describing the company/product, and labels for the news viewpoint.

According to the dataset’s conceptualization, native advertising is sponsored media that is intended to merge inconspicuously with editorial content, making it challenging for viewers to identify it as such. We used sentiment, persuasiveness, product or company promotion, and perspective—four implicit traits that set native advertising apart from regular news reporting—to define it. First, Native ads typically steer clear of critical or negative framing in favor of a positive or neutral tone. This is different from traditional journalism, which frequently offers a variety of opinions, including critical ones. An article advertising a new smartphone, for instance, can highlight its innovative features and advantages for users while downplaying any potential disadvantages, including its high cost or technical constraints. Secondly, native ads are compelling by nature. They are written with the intention of influencing the reader’s thoughts and behaviours, such as buying a product or recommending a service. Although it is frequently subtle, this persuasiveness is woven throughout the story of the article. An post about an eco-friendly product, for example, might emphasize how simple it is to use and how it benefits the environment, making readers think it’s a better option than traditional products.

Third, native advertisements frequently concentrate on favorably promoting a certain brand, business, or service. Since it is usually weaved within a larger narrative, the promotional goal is not always obvious. For example, an article about sustainable energy solutions could portray a certain energy company as a pioneer in the industry, quietly highlighting the business without overtly advertising it. Finally, native advertisements usually showcase information from a single viewpoint that is in line with the objectives of the company or item being advertised. Native advertisements offer a biased narrative that supports the advertising message, in contrast to balanced journalism, which presents a variety of perspectives or counterarguments. An article about a financial institution’s new savings program, for instance, might focus on the advantages without discussing any possible drawbacks or contrasting it with other offerings. The theoretical foundation for recognizing native advertising in our dataset is based on these four attributes: favorable promotion, persuasive intent, positive or neutral sentiment, and single perspective. To give an example, consider the article ‘How XYZ Company is Revolutionizing the Tech Industry,’ which skillfully blends editorial and promotional content by showcasing the company’s accomplishments without mentioning any obstacles or rivals. This conceptual framework served as the foundation for our data annotation procedure, guaranteeing that the dataset captures the subtle ways in which native ads imitate news items. This ensures that native ad detection research has a strong foundation.

In constructing our dataset, we have based the labeling on the implicit characteristics of native advertisements. Native advertising arose due to inadequate sponsorship transparency in media, aiming to integrate ads smoothly into content but often criticized for insufficient disclosure^3^. Native ads often blend seamlessly into regular news sections, mimicking the style and content of genuine news articles, which necessitates a nuanced approach to their detection and classification in our dataset.

**Fig. 1:**
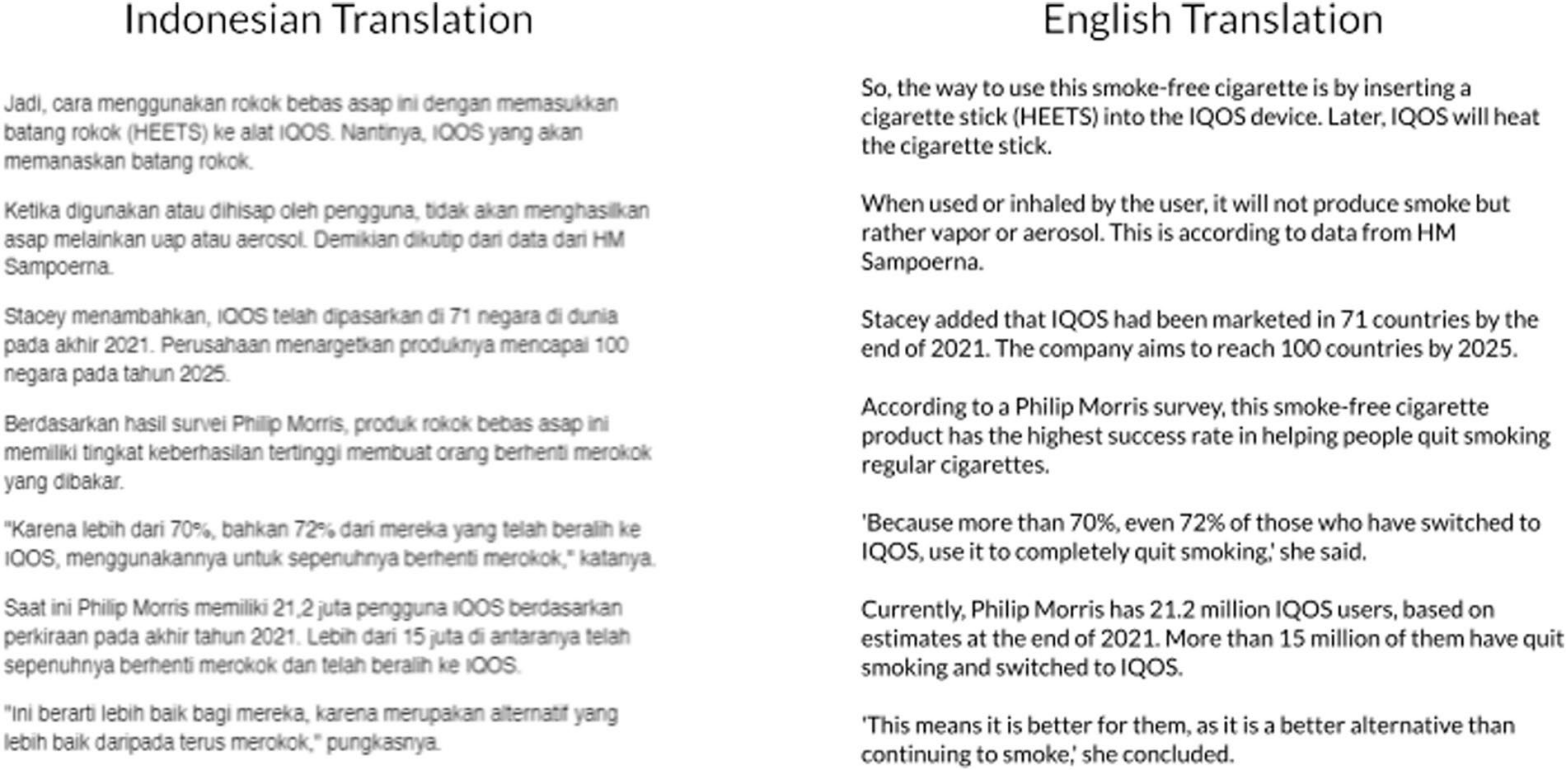
Example content for sentiment label.

**Fig. 2:**
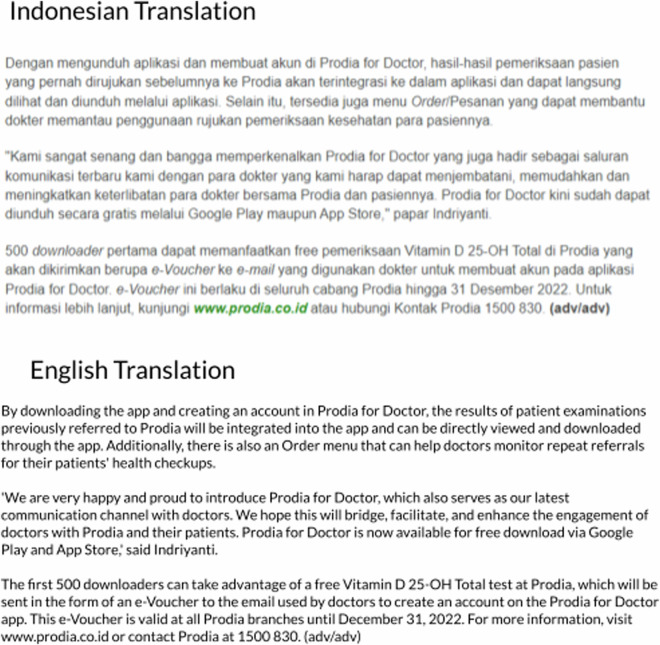
Example content for persuasive news.

Based on the descriptions provided, it’s clear that each image illustrates a different implicit characteristic of native advertising within the content. Figure 1 illustrates a positive sentiment towards smoke-free cigarettes, portraying them as beneficial and a viable alternative for smokers. Despite the generally negative connotation associated with smoking and its health risks, the news content in this image presents cigarettes in a positive light. This exemplifies native ads content that carries a positive sentiment. Figure 2 discusses the “Prodia for Doctor” app, highlighting its benefits in the healthcare sector. However, the presence of a persuasive sentence at the end of the paragraph, urging readers to download the app, showcases the persuasive characteristic typical of native advertising.

**Fig. 3:**
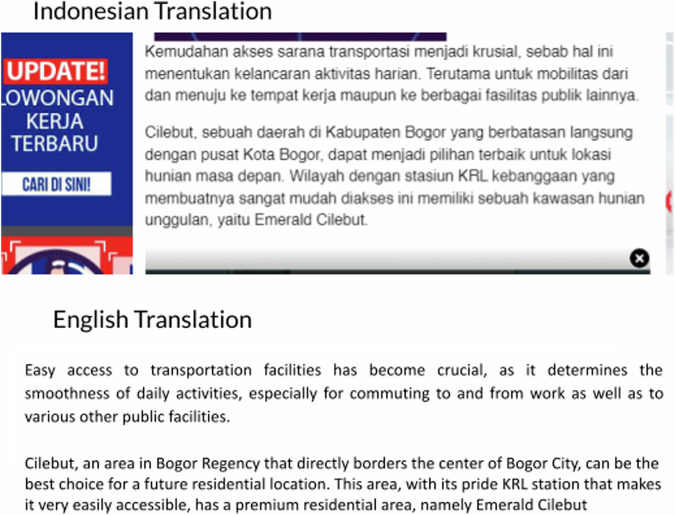
Example content for depicting a company or product.

**Fig. 4:**
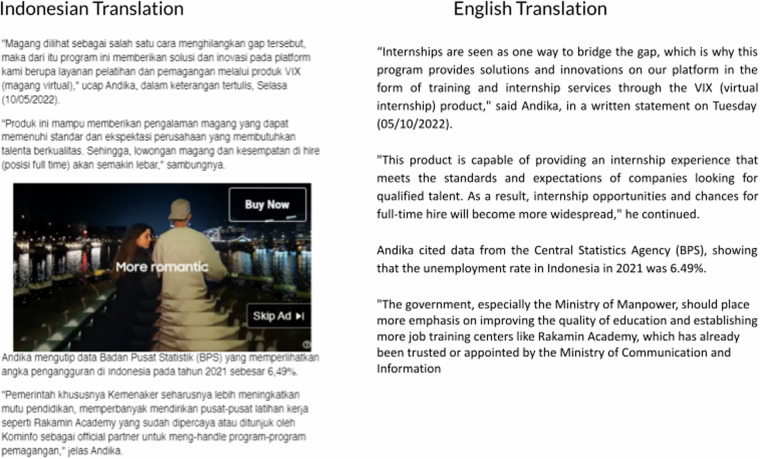
Example content for one perspective.

Figure 3 describes a comfortable residential area with easy access, focusing on one particular housing cluster presented in a favorable manner. This indicates the native ad’s characteristic of depicting a company or product advantageously. Figure 4 focuses on internship news but interviews only one perspective. This showcases the characteristic of presenting a single viewpoint, a common trait in native advertising to streamline the narrative in favor of the advertised product or service. These examples serve as the foundation for constructing a dataset that identifies the implicit characteristics of native ads. This dataset aims to facilitate the detection of such ads on Indonesian electronic news portals by annotating and validating these implicit traits alongside the explicit mention of native advertising.

**Fig. 5:**
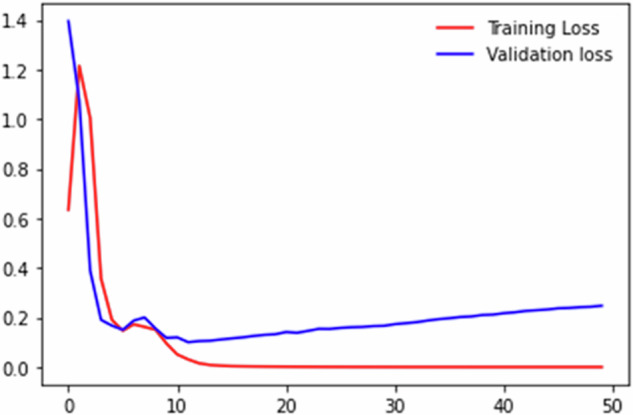
Training and validation loss of BERT-BiLSTM.

This dataset was constructed through a process of web scraping, data annotation, and expert validation. The dataset has undergone testing for detecting native ads and persuasive news through the application of deep learning and ensemble learning methods. In the initial experiment^12^, the dataset was segmented into training and test sets with a division ratio of 70% to 30%. We utilized 200 data points, dividing them into 140 for training and 60 for testing. The dataset was evaluated to identify the best methods for word embedding (BERT, FastText, GloVe) and deep learning (BiLSTM, CNN, LSTM). Preprocessing was performed on the dataset, followed by the application of word embedding techniques to obtain word vectors. The final step involved developing a model using deep learning. The findings indicated that the accuracy performance of the BERT-BiLSTM model reached 95%. Although the BiLSTM obtained promising results, the model still experienced issues with overfitting. This is evident in Fig. 5, which shows the validation loss diverging from the training loss.

**Fig. 6:**
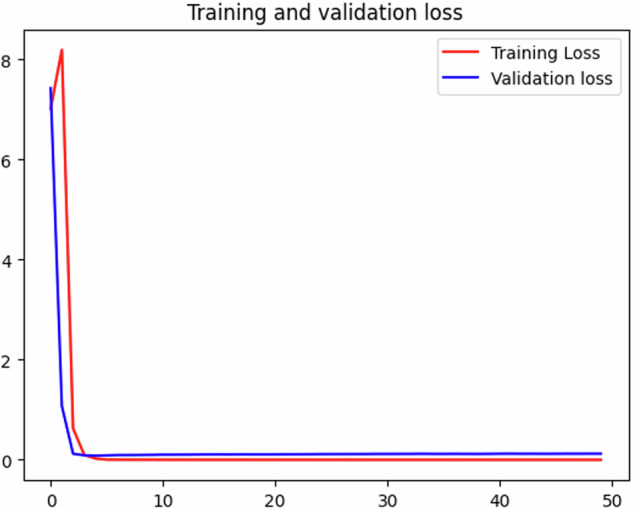
Validation loss and training loss of BiLSTM-CNN with attention mechanism and parameter tuning.

In the second experiment^13^, we employed the same data but opted for a different classification model. Given that the first experiment yielded promising results, but the model suffered from overfitting, we aimed to address this issue. We experimented with an ensemble method, combining two deep learning approaches. The result was that the BiLSTM-CNN model achieved a lower score than the standalone BiLSTM, with an accuracy of 92.5%. We then introduced an attention mechanism and conducted parameter tuning on the model. Ultimately, the BiLSTM-CNN model achieved an accuracy of 95% and resolved the overfitting problem. Figure 6 illustrates that the validation loss and training loss are nearly parallel, indicating a significant improvement in model performance and generalization.

In our third experiment^14^, we concentrated on identifying certain implicit characteristics of native advertisements, particularly focusing on the identification of persuasive news content. Utilizing a dataset containing 1,708 records, our annotators found only a limited number of persuasive sentences within each piece of extensive news content. Consequently, we opted to employ a Tag Summarization technique to distill the core elements of the news. We adopted a hybrid methodology combining Text Summarization and Deep Learning techniques. The results showed that our TextRank-BERT-BiLSTM model achieved a 95% accuracy rate. Table 1 demonstrates that the methods we utilized surpassed other NLP techniques, proving the effectiveness of our approach in accurately identifying persuasive content in news articles.

**Table 1:** State-of-the-art comparison methods.

Method	Accuracy	Precision	Recall	F1 Score
BERT (Transformer)	0.92	0.95	0.9	0.93
BiLSTM-CNN-Attention (Attention)	0.9	0.91	0.89	0.9
BiLSTM-LSTM (Ensemble Model)	0.57	0.61	0.53	0.45
MLP (Feed Forward)	0.88	0.88	0.88	0.88
TextRank-BERT-BiLSTM	0.95	0.95	0.95	0.95

Each annotated news content in this dataset exhibits high variability, as each piece of news is simultaneously assigned five labels. This high variation in news content makes the dataset particularly challenging for detecting and recognizing native ads. The annotation process was conducted manually by experts. These experts read each piece of content and then assigned five labels accordingly. This high level of accuracy demonstrates the efficacy of the dataset and the chosen deep learning architectures in identifying native advertising within news content, despite the inherent challenges posed by the dataset’s complexity and variability.

## Methods

**Fig. 7:**
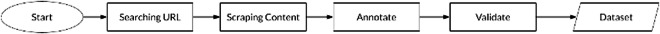
Schematic representation of the processing steps involved in creating the native ads dataset.

**Fig. 8:**
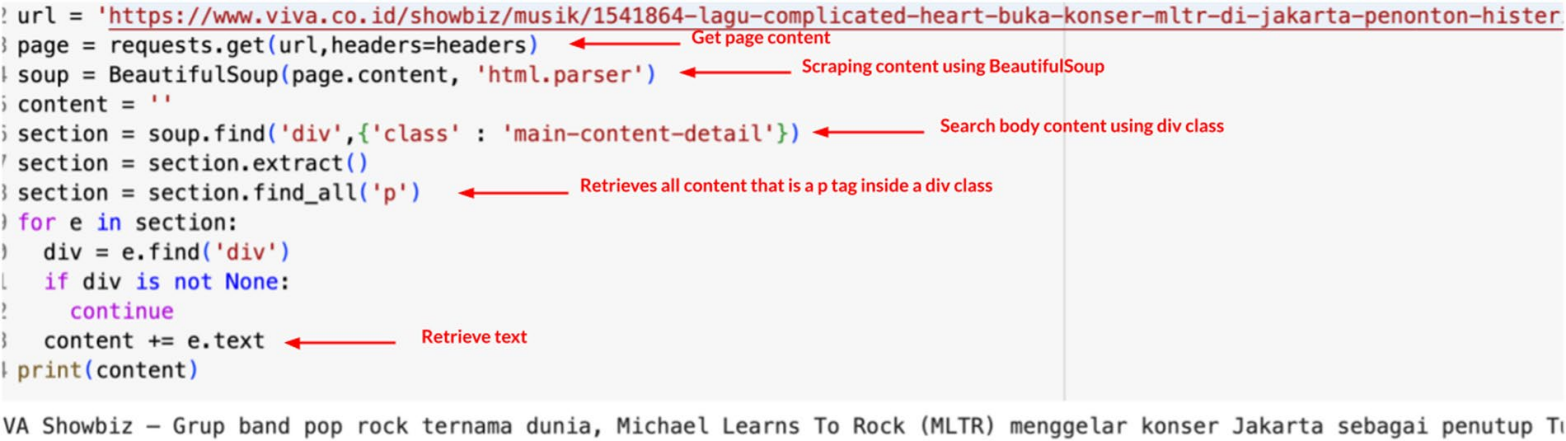
Example code for scraping content.

The dataset was constructed through a four-stage process depicted in Fig. 7. It started with the extraction of URLs using the BeautifulSoup library to scrape data from six electronic news portals. BeautifulSoup has strengths in constructing a parse tree from HTML and providing straightforward methods for traversing, searching, and modifying the parse tree^15^. The second stage focused on retrieving news content using the same scraping technique. The URLs obtained in the first step were processed to extract text data, excluding images. This resulted in 12,088 contents, with each news content averaging between 10 to 20 paragraphs. Table 2 presents the comprehensive statistics of the dataset, where efforts have been made to balance the quantities of native ads and news articles to prevent overfitting due to imbalanced data. The dataset includes an equal number of native ads and news articles, with 6,044 entries each. News articles and native ads are evenly distributed throughout the collection. We purposefully balanced the dataset to guarantee the classification model’s efficacy, even though normal news items actually outweigh native ads in the majority of media sites. Classifiers have a tendency to favor the majority class in imbalanced datasets, which may lead to subpar performance when detecting native ads. We used a balancing technique when building the dataset to avoid this bias and improve the model’s capacity to correctly differentiate between news and native advertisements. It should be mentioned that the 50/50 ratio only represents the dataset utilized in this study and does not accurately reflect how native ads are distributed throughout Indonesia’s media ecosystem. The balancing was done solely to facilitate accurate machine learning analysis and classification. The news categories covered are Economy, Lifestyle, Entertainment, International, National, Others, Automotive, Education, Sports, and Technology. Table 3 details the category distribution for both native ads and news labels. Figure 8 explains the code for scraping one of the contents. Detail each step of scraping content in the following sections.

**Table 2:** The statistics of the native ads dataset.

News Portal	Total Data	Native Ads Label	News Label
Electronic News Portal 1	1415	521	894
Electronic News Portal 2	4026	2869	1157
Electronic News Portal 3	1290	220	1070
Electronic News Portal 4	2384	1221	1163
Electronic News Portal 5	2006	826	1180
Electronic News Portal 6	967	33	934

**Table 3:** Distribution of categories for the native ads and news labels.

Category	Total Data	Native Ads Label	News Label
Economy	1914	1349	565
Lifestyle	1807	415	1392
Entertainment	635	128	507
International	359	90	269
National	1262	595	667
Others	3565	2882	683
Automotive	474	106	368
Education	274	70	204
Sports	823	69	754
Technology	975	340	635

### Get page content

The ‘requests.get‘ method is specifically used to send a GET request to a server, which is the most common HTTP request method. It is designed to request data from a specified resource, without the intention of affecting the resource’s state. This contrasts with other HTTP methods such as POST, which are used to submit data to be processed to a specified resource, potentially leading to changes in server state or side effects.

### Scraping content using BeautifulSoup

The use of BeautifulSoup(page.content, ‘html.parser’) within the Python ecosystem is a significant approach in web data processing and analysis, particularly in the context of parsing HTML and XML documents. The use of page.content as the first argument in the BeautifulSoup function indicates that the parsed data is the raw content of a web page that has been fetched, typically through an HTTP request using libraries such as requests. This content, which is generally in byte form, is then transformed by BeautifulSoup into a data structure that can be easily navigated and searched.

### Search body content using div class

The use of the method soup.find(‘div’, {‘class’: ‘main-content-detail’}) is a highly valuable technique for extracting specific information from HTML documents. This technique is part of the BeautifulSoup library in Python, which is widely recognized in both academic and industry communities for its ability to parse and manipulate web data in an efficient and intuitive way. The find method from the BeautifulSoup object allows researchers to selectively access specific parts of the HTML structure based on defined criteria, in this case, the div element with the class main-content-detail.

### Retrieves all content

The use of the method section.find_all(‘p’) in the BeautifulSoup Python library reflects an advanced technique in the extraction and analysis of web content. This method leverages BeautifulSoup’s capability to perform deep searches within HTML or XML documents, allowing researchers to systematically identify and collect all paragraph ( < p > ) elements contained within a specific section of a web page. This approach is highly relevant in studies aimed at analyzing text or narrative content from online sources, given that the < p > element is often used to present main text blocks within the structure of web pages.

### Retrieve text

The use of the expression e.text within the context of the BeautifulSoup Python library is a fundamental technique for extracting text from web elements. This expression represents an operation that retrieves the textual content from an identified or selected element within the structure of an HTML or XML document. It serves as an efficient method for obtaining raw data from web elements, which can then be further analyzed for various research purposes.

The third stage entailed data annotation. The news content was assigned 5 labels. However, according to experts, not all sentences within a single news content contain native ads indicators; only a few sentences might suggest that the news falls under native ads. Therefore, to determine the labels, experts were involved in assigning these 5 labels. News annotation was performed to label news used as ground truth. Annotating native ads on electronic news portals yielded a new native ads dataset for identifying native ads.

The final stage was data validation. Based on our experimental results, this dataset was capable of achieving performance up to 95%. This structured approach to dataset development ensures a comprehensive and reliable resource for identifying native advertising within electronic news content, leveraging expert input for accurate annotation and validation to enhance the dataset’s effectiveness in real-world applications.

## Data Records

The Native Ads Dataset is freely accessible to researchers via figshare^16^. Our study’s goal was to choose news stories from the data collection period that were most pertinent to the current state of affairs. Relevance was determined by concentrating on news topics that were hot and talked about at the time, like politics, economics, and health, which were especially prevalent in Indonesian media between February and June of 2022. The data was gathered between February 2022 and June 2022 because that is when I started my doctoral studies and my data collection process. Practical considerations, such as the tools and resources available when I began my investigation, led to the selection of the timeframe. The annotation procedure was very lengthy because the dataset required to be meticulously and comprehensively labeled by skilled annotators, guaranteeing high accuracy and validation at every turn. This painstaking procedure lasted until the middle of 2024. The complexity and duration of the annotation process would have been greatly enhanced, possibly leading to delays, if the data gathering period had been extended past these months. In order to balance the viability of the annotation process with the timely completion of the study, we concentrated on extending this collection period. We explored various news categories and selected the most recent news relevant to the current situation.

Our Native Advertisement Dataset served as the benchmark for subsequent automated classification attempts, while expert annotators played a key role in establishing the gold standard for manual categorization. The procedure of recognizing native advertisements was based on a rigorous set of rules, including the use of a predefined codebook, annotator training, and a systematic evaluation of intercoder reliability. Before beginning the annotation process, each annotator underwent a rigorous training program. After an overview of the theoretical foundations of native advertising, annotators engaged in hands-on activities where they practiced coding a variety of news articles under the guidance of subject-matter specialists. During this phase, the annotators received feedback to correct any mistakes or ambiguities and enhance their understanding of the codebook. The training also included many calibration sessions to reduce subjectivity and synchronize the annotators’ evaluations.

The data annotation process was conducted and validated by experts with significant experience in media analysis and advertising. Initially, a set of preliminary labels was established based on a review of the content and the research objectives, which aimed to classify the data into five distinct categories. To ensure the appropriateness and accuracy of these labels, we employed a multi-step validation process. First, the annotators independently reviewed a subset of the dataset to apply the labels and assess whether the initial categories were comprehensive enough to capture all relevant distinctions. Following this, we conducted a series of iterative feedback sessions where the annotators discussed any ambiguities or inconsistencies in the labeling process. These sessions allowed for adjustments to the labeling guidelines to ensure clarity and precision. Finally, a consensus was reached among the annotators regarding the definitions of each label, and the full dataset was annotated accordingly. The final labels were validated through cross-checks and random sampling to confirm that the annotations were consistent and accurately reflected the content. This thorough process ensured that the labels were both relevant and reliably applied throughout the dataset.

The five main classifications that make up the Native Ads Dataset each reflect a unique feature of the news articles: sentiment, persuasive, company or product description, news perspective, and native ads. The format of the annotation file is as follows: <id> <date> <content> <positive-label> <persuasive-label> <product-label> <perspective-label> <native-ads-label>. Every article has a unique identity in the <id> column, which makes it possible to refer to each entry separately. The article’s publication date, which is crucial for temporal analysis and comprehending trends over time, is entered in the <date> field. The news article’s entire body, which is the main text used for classification, is contained in the <content> column.

Additionally, the dataset has a number of significant annotation labels. The article’s emotional tone is captured by the <positive-label> or sentiment label, which classifies it as either positive, neutral, or negative. This sheds light on the article’s overall emotional position. The article’s level of persuasiveness is indicated by the <persuasive-label>, which also marks the article’s promotional intent by noting if the content aims to persuade readers to buy a product, service, or point of view. The <product-label> allows the identification of commercial entities featured in the content by providing a description of the firm, brand, or product mentioned in the article, if relevant. The article’s journalistic perspective is reflected in the <perspective-label >, which indicates whether the tone is more subjective and opinion-based or objective and factual. The <native-ads-label >clearly indicates if the piece is native advertising, which combines editorial and advertising information in a way that lessens the prominence of the promotional content.

We purposefully left out columns with media source information during the data collection process. Legal factors, specifically those pertaining to copyright laws and privacy standards that applied to the platforms where the news information was first published, were taken into account while making this judgment. A thorough examination of each piece’s editorial character as well as any possible promotional content is made possible by the organized framework, which guarantees that every article is thoroughly annotated. A variety of research and machine learning projects centered on native advertisement recognition can benefit from the dataset’s inclusion of numerous labels, which enables nuanced assessments of the intersection between news and advertising.

## Technical Validation

Expert assessment and the use of a Bidirectional Long Short-Term Memory (BiLSTM) deep learning model were the two main approaches used for the data validation procedure. Domain experts with experience in media and advertising analysis carried out the expert review. Following predetermined parameters that centered on traits like integration within editorial material, promotional intent, and subtle advertising indicators, these experts manually annotated a subset of the dataset to detect native advertisements. In order to maintain consistency and reduce any potential biases, the procedure involves several rounds of annotation, feedback, and improvement. The experts reached a consensus to settle any disagreements over annotations, guaranteeing the accuracy and dependability of the finished dataset. Throughout this process, no notable departures from the established parameters were noticed.

The annotated dataset was further validated using a BiLSTM model in addition to expert review. The model was evaluated using common performance metrics like accuracy, precision, recall, and F1-score after being trained on the expert-labeled data. This made it possible for us to assess the model’s functionality and guarantee the caliber of the annotations. There were no discernible differences between the expert-labeled ground truth and the model’s predictions, and the validation procedure verified that the annotations were consistent. When combined, these two validation techniques—deep learning and expert evaluation—offered a thorough and exacting mechanism for guaranteeing the dataset’s quality and dependability.

## Usage Notes

The native ads dataset was published in a single file with an Excel (xlsx) format named native_ads_dataset.xlsx.

## Data Availability

The native ads dataset is available on the Figshare repository^16^ and the scraping code is available on a GitHub repository (https://github.com/brianrizqi/news-scraping). The data scraping was performed using the BeautifulSoup library in Python, and the code is available online^17^.
